# Spatiotemporal Control of GPR37 Signaling and Its Behavioral Effects by Optogenetics

**DOI:** 10.3389/fnmol.2018.00095

**Published:** 2018-03-28

**Authors:** Wu Zheng, Jianhong Zhou, Yanan Luan, Jianglan Yang, Yuanyuan Ge, Muran Wang, Beibei Wu, Zhongnan Wu, Xingjun Chen, Fei Li, Zhihui Li, Sergii Vakal, Wei Guo, Jiang-Fan Chen

**Affiliations:** ^1^Molecular Neuropharmacology Laboratory, School of Optometry and Ophthalmology and Eye Hospital, Wenzhou Medical University, Wenzhou, China; ^2^State Key Laboratory of Optometry & Vision Science, Wenzhou, China; ^3^Department of Neurology, Boston University School of Medicine, Boston University, Boston, MA, United States

**Keywords:** GPCRs, orphan GPCRs, opto-GPR37, channelrhodopsin, optogenetics

## Abstract

Despite the progress in deorphanization of G Protein-Coupled Receptors (GPCRs), ≈100 GPCRs are still classified as orphan receptors without identified endogenous ligands and with unknown physiological functions. The lack of endogenous ligands triggering GPCR signaling has hampered the study of orphan GPCR functions. Using GPR37 as an example, we provide here the first demonstration of the channelrhodopsin 2 (ChR2)-GPCR approach to bypass the endogenous ligand and selectively activate the orphan GPCR signal by optogenetics. Inspired by the opto-XR approach, we designed the ChR2-GPR37 chimera, in which the corresponding parts of GPR37 replaced the intracellular portions of ChR2. We showed that optogenetic activation of ChR2/opto-GPR37 elicited specific GPR37 signaling, as evidenced by reduced cAMP level, enhanced ERK phosphorylation and increased motor activity, confirming the specificity of opto-GPR37 signaling. Besides, optogenetic activation of opto-GPR37 uncovered novel aspects of GPR37 signaling (such as IP-3 signaling) and anxiety-related behavior. Optogenetic activation of opto-GPR37 permits the causal analysis of GPR37 activity in the defined cells and behavioral responses of freely moving animals. Importantly, given the evolutionarily conserved seven-helix transmembrane structures of ChR2 and orphan GPCRs, we propose that opto-GPR37 approach can be readily applied to other orphan GPCRs for their deorphanization in freely moving animals.

## Introduction

G Protein-Coupled Receptors (GPCRs) are the most abundant family of cell membrane proteins in the human genome, which regulates almost all cellular signaling pathways and plays a vital role in numerous physiological and pathological processes (Bockaert and Pin, 1999; Rosenbaum et al., 2009). To date, the drugs targeting GPCRs account for about a third of the total medicines in the market, which makes them excellent potential drug targets (Santos et al., 2017). Orphan GPCRs play a crucial role in sleep rhythm, blood pressure, hormone homeostasis, tumor growth and nervous system disorders (Lin et al., 1999; Marazziti et al., 2004; Civelli, 2012; Tang et al., 2012; Nath et al., 2017). Despite extensive research over the last three decades, approximately 100 GPCRs remain orphan receptors with unknown and unmatched endogenous ligands (Davenport et al., 2013).

Among the orphan GPCRs, GPR37, a parkin-associated endothelin-like receptor, is an orphan receptor linked with autosomal recessive juvenile Parkinson’s disease (Yang et al., 2003). GPR37 is highly expressed in brain regions such as corpus callosum, caudate nucleus, putamen, substantia nigra, hippocampus and cerebellum (Donohue et al., 1998; Lopes et al., 2015). GPR37 is emerging as one of the promising targets for Parkinson’s disease therapy from several lines of evidence: (I) GPR37 was accumulated and upregulated in Lewy bodies of PD patients (Murakami et al., 2004; Wang et al., 2008; Leinartaité and Svenningsson, 2017). (II) Genetic knockout (KO) of GPR37 showed altered dopamine (DA) signaling, MPTP resistance, decreased locomotor activity, and defect of motor coordination (Marazziti et al., 2004). (III) Overexpression of GPR37 in the nigrostriatal region of rats resulted in pathologic changes common to Parkinson’s disease (Dusonchet et al., 2009). (IV) GPR37 KO was associated with demyelination, depression, anxiety and conditioned place preference (Marazziti et al., 2011; Liu et al., 2014; Lopes et al., 2015; Wang et al., 2016; Rial et al., 2017; Smith et al., 2017). However, the elucidation of GPR37 and other orphan GPCR functions has been limited to the transgenic overexpression and KO/knockdown (KD) of target genes (in large part due to the unknown endogenous ligands). The putative ligand head activator (HA) has been proposed based on its high-affinity for GPR37 (Rezgaoui et al., 2006), but evidence for an HA ortholog in vertebrates is lacking (Meyer et al., 2013). Furthermore, prosaptide and prosaposin have been shown to bind to GPR37 and produced a significant increase in p-ERK level and inhibition of cAMP *in vitro*, indicating G_i_ signaling of GPR37. However, this effect of prosaptide has not been widely studied (Meyer et al., 2013; Lundius et al., 2014) and specific endogenous ligand for GPR37 remains unclear. Unknown ligands hamper functional analysis of orphan GPCRs because neither overexpression nor gene silencing can trigger the downstream signaling and produce activation of orphan GPCRs induced by ligands in specific temporal patterns. New selective approaches to mimic endogenous GPR37 kinetics with spatiotemporal resolution are essential to unravel biological functions of orphan GPCRs.

To overcome this significant limitation in this study, we proposed a novel opto-XR approach to by-pass the endogenous ligand and trigger orphan GPCR signaling by light for probing GPR37 functions in intact animals. This approach is based on the “opto-XR” that was first demonstrated in the cultured cells (Kim et al., 2005) and implemented recently in intact animals to study the function of GPCRs (opto-α_1_AR and opto-β_2_AR) with high spatiotemporal precision (Airan et al., 2009). Opto-XR is designed to replace the intracellular loop (IL) sequences of rhodopsin with the sequences of targeted GPCR. This chimeric opto-XR protein can selectively activate GPCR signaling pathways in response to light and simulate the pharmacological effects of ligands. For example, light activation of opto-MOR in GABAergic neurons altered place preference behavior (Siuda et al., 2015). Similarly, activation of opto-mGluR6 in ON-bipolar cell restored vision (van Wyk et al., 2015). We have also recently successfully developed opto-A_2A_R to demonstrate that light activation of opto-A_2A_R signaling triggers cAMP accumulation, ERK phosphorylation, CREB phosphorylation, induction of LTP in the hippocampus and impairs memory (Li et al., 2015, 2016).

Inspired by the opto-XR approach and by successful adaption of opto-A_2A_R, we reasoned that GPR37 and many other orphan GPCRs share conserved structure of seven-helix transmembrane domains (TM) among various animal species and, thus, can be used to define the IL of orphan GPCRs. Also, to improve its responsiveness to light, we channelrhodopsin-2 (ChR2; Inaguma et al., 2015) to replace rhodopsin as the backbone for the extracellular and transmembrane portion of opto-GPR37. As there is little sequence homology between channelrhodopsin and orphan GPCRs, we used the defined the N-terminus, extracellular (EL1–EL3) and TM (TM1–TM7) of channelrhodopsin family (Inaguma et al., 2015). We also identified the ILs (IL1–IL3) and C-terminus of GPR37 by sequence alignment of GPR37 in different species including *Homo sapiens*, *Mus musculus*, *Callorhinchus milii*, *Latimeria chalumnae* (*lch*), *Xenopus tropicalis* and *Gallus gallus*). We then created an opto-GPR37 chimera by fusing the N-terminus, extracellular and transmembrane portions of channelrhodopsin with the ILs and C-terminus of GPR37. This opto-GPR37 approach allowed us to bypass endogenous ligands and directly activate orphan GPCR signaling by the light. GPR37 is highly expressed in the striatum and implicated in PD pathogenesis with striatum as a critical locus. Given that striatum also expresses a high level of receptors for neurotransmitters and neuromodulators (such as DA receptor and the adenosine A_2A_ receptor) with a known interaction with GPR37 (Morató et al., 2017), we targeted the striatum for analysis of opto-GPR37 signaling and behavioral responses. We used GPR37 as an example to illustrate the feasibility and specificity of ChR2-GPR37 approach for probing orphan GPCR signaling and biochemical and behavioral responses. In response to blue light, GPR37 signaling was elicited, and subsequent biochemical (i.e., ERK phosphorylation, DARPP-32 phosphorylation, c-Fos expression) and behavioral (i.e., motor and anxiety-like) responses were assessed. Our results suggest that ChR2-GPR37 is a useful tool to probe GPR37 function in a specific and temporally precise manner in freely moving animals. We propose that ChR2-orphan GPCR represents a novel approach to provide new insights on functions of orphan GPCRs in intact animals.

## Materials and Methods

### Animals

This study involved animals but not human participants. All animals were handled following the protocols approved by the Institutional Ethics Committee for Animal Use in Research and Education at Wenzhou Medical University, China. C57/B6 3-month-old male mice with the weight from 25 to 30 g were randomly divided into two groups. Mice were housed in groups of 3–5 per cage on a standard 12 h light/12 h dark cycle.

### Design and Construction of the opto-GPR37 Vector

To enhance light responsiveness, we used ChR2 to replace rhodopsin as the backbone for the extracellular and transmembrane parts of opto-GPR37. Because of low sequence homology between the selected orphan GPCR and channelrhodopsin, we individually defined the IL of GPR37 (*Homo sapiens*, NP_005293.1) by alignment of GPR37 sequence from different species that share conserved structure of seven TM (cd15127: 7tmA_GPR37). We then defined the extracellular and TM of channelrhodopsin-2 (*synthetic construct*, ABO64386.1) by sequence alignment of channelrhodopsin family, as has been reported before (Inaguma et al., 2015). Then, we constructed a fusion gene encoding a chimera (opto-GPR37) by replacing the ILs 1, 2 and 3 and the C terminus of ChR2 with those of GPR37. Last, codon-optimized sequences of opto-GPR37 were fused with the N terminus of mCherry (with its start codon deleted) with a linker (5′-GCGGCCGCC-3′) for fluorescence detection of opto-GPR37 in cells and tissues. The opto-GPR37 construct was cloned into a pcDNA3.1 vector (EF1α-opto-GPR37-mCherry).

### Viral Production

Recombinant adeno-associated viral (AAV) vectors were constructed with a transgene cassette encoding Synapsin promoter by cloning the opto-GPR37 into pHBAAV-hsyn-MCS-T2A-mCherry (HANBIO) using *Kpn*I/*Bam*HI. Viral particles were packaged and purified by HANBIO Biotechnology (Shanghai, China) for serotype 9, and the titers were 1.2 × 10^12^ particles per ml. The AAV2/9-Syn-mCherry was purchased from Hanbio Biotechnology as the “control” virus with a titer of 1.4 × 10^12^ particles per ml.

### Transfection and Detection of an opto-GPR37 Signal *in Vitro*

HEK293T cells were transfected using Lipofectamine 2000 (Invitrogen, Cat #11668-019) according to the protocol. To study opto-GPR37 signaling, we added and incubated all-*trans*-retinal (Sigma, Cat#R2500) at 37°C for overnight. HEK293 cells were kept in 384-well plates and illuminated (Aurora-220-473, NEWDOON, Hangzhou, China) for 60 s at 37°C (473 nm, 4 wells/region, 3 mW, 20 ms, 20 Hz) with a standard distance of illumination (2 cm) following the protocol of our previous study with opto-A_2A_R (Li et al., 2015). Ten minutes after light stimulation, the cells were lysed to analyze cAMP content using cAMP HTRF^®^ Assay kit (Cisbio Assay, Cat# 62AM4PEC). The level of p-ERK was measured by AlphaScreen® SureFire® p-ERK 1/2 (Thr202/Tyr204) Kit (Perkin Elmer, Cat# TGRES500) and IP-1 (inositol-1-phosphate) content was analyzed by HTRF IP-1 assay kit (Cisbio Assay, Cat# 62IPAPEC). To study kinetic activation of p-ERK *in vitro*, we stimulated the cells of the opto-GPR37 group with light for 60 s. At the various time points (0, 5, 15, 30, 60 min) after the 60-s light stimulation, the cells were collected and lysed to analyze p-ERK by AlphaScreen^®^ SureFire^®^ p-ERK 1/2 (Thr202/Tyr204) Kit (Perkin Elmer, Cat# TGRES500).

### Surgical Procedures

Three-month-old C57/B6 male mice were injected with 3.6% chloral hydrate (0.1 ml/10 g) and placed in a stereotactic frame. The scalp was shaved, ophthalmic ointment (oculentum aureomycin) was applied to the eyes, and 75% ethanol was used to sterilize the surgical area. Two-hundred nanoliter of virus (AAV2/9-syn-opto-GPR37-mCherry or AAV2/9-syn-mCherry) was injected into the dorsal medial striatum (DMS; AP: 0.98, ML: −1.3, DV: 2.6). A unilateral optical fiber (ferrule O.D: 2.5 mm, fiber core: 200 μm, length: 3.5 mm) was implanted 0.05 mm above the injection site (AP: 0.98, ML: −1.3, DV: 2.55). Animals were allowed to recover for 3 weeks before the behavioral and histochemical analyses. Mice were excluded from further analysis if their body weight was out of normal range (20–35 g) after recovery from the surgery.

### Immunohistochemistry and TUNEL Staining

DARPP-32 (Thr75) staining: light stimulation (473 nm, 20 ms, 20 Hz, 10 mW) for 10 min, then after 30 min mice were perfused with ice-cold 4% paraformaldehyde in PBS (pH = 7.4); c-Fos staining: light stimulation for 15 min, then after 90 min mice were perfused with ice-cold 4% paraformaldehyde in PBS (pH = 7.4). Brains were postfixed, and coronal sections with 20 μm were cut for immunohistochemistry. Free-floating sections were washed in PBS and then incubated for 30 min in 0.3% Triton X-100 and 3% serum. Primary antibody incubations were conducted overnight in 0.01% Triton X-100 and 3% normal rabbit serum for DARPP-32 (Thr75; CST; 1:300) or c-Fos (EMD Millipore; 1:1000). Sections were then washed with PBS and incubated for 2 h at room temperature with Alexa Fluor^®^ 488 sary antibodies (Abcam; 1:400). Slices were then washed and mounted on slides with VECTASHIELD mounting media. Images were acquired with a two-photon confocal microscope (LSM880, Zeiss, Germany). Three successive images from microscope field under the 20× objective were acquired within a 500-μm region beneath the injection site, and the positive signals per field were counted, and signals from three representative field were averaged for each animal.

To identify the potential damage induced by light stimulation (473 nm, 10 mW, 20 ms, 20 Hz), the TdT-mediated dUTP Nick-End Labeling (TUNEL) staining was performed by using a commercial *in situ* cell death detection kit and following the manufacturer’s manual (Roche Diagnostics, Basel, Switzerland). Briefly, the injected mice were subjected to the light illumination (473 nm, 10 mW, 20 ms, 20 Hz) for 30 min. Then the mice were anesthetized using 3.6% chloral hydrate and then decapitated. The striatal sections were permeabilized, and antigen retrieval was performed with 0.1% sodium citrate buffer solution with 0.1% Triton X-100 for 5 min at 4°C. After three-time washing, the sections were incubated in TUNEL reaction solutions for 1 h at 37°C and washed. The fluorescent mounting medium was applied to the sections. The positive control was also performed to ensure the reliability of results by adding DNase I reaction solution (incubation for 20 min) on the sections of AAV-2/9-Syn-mCherry to induce the DNA break. Fluorescent double staining was visualized by confocal microscopy (LSM880, Zeiss).

### Western Blotting and Real-Time Quantitative PCR

After the light stimulation (473 nm, 20 ms, 20 Hz, 10 mW) for 10 min, we injected 100 nl of trypan blue (0.4%, Sigma, Amresco) in DMS (AP: 0.98, ML: −1.3, DV: 2.6) to the same site where we have injected the virus. After 5 min the mice were anesthetized and decapitated to collect the samples of the blue area. Samples were prepared for RNA or protein isolation. Tissue was homogenized in 1 ml RIPA (50 mM Tris HCl, pH = 8.0, 150 mM NaCl, 1% NP-40, 0.5% sodium deoxycholate, 0.1% SDS) buffer with a hand homogenizer (Sigma), incubated on ice for 15 min, and rotated at 4°C for 30 min. Cell debris was isolated and discarded by centrifugation at 14,000 rpm for 10 min. Lysates were quantitated using a nanodrop, and protein was loaded in a 12% acrylamide gels. Protein was transferred from acrylamide gels to PVDF membranes (Invitrogen) at 100 V for 120 min. Membranes were blocked using bovine serum albumin (5% w/v). Membranes were incubated with primary antibodies overnight at 4°C and secondary antibodies at room temperature for 90 min. Primary antibodies were anti-p-ERK (CST; 1:1000) or anti-ERK (CST; 1:1000). The secondary antibody was IRDye^®^ 800CW (LI-COR; 1:5000). Signal intensities were calculated using ImageJ software and normalized to values of ERK.

The mice were anesthetized using 3.6% chloral hydrate and decapitated. HEK293 cells and DMS from these injected mice were collected and frozen with liquid nitrogen, and then the samples were stored at −80°C. The total RNA was extracted according to a TRIzol method. Concentration and purity of total RNA were detected by NanoDrop ND 1000 (Thermo Scientific). PrimeScript RT Master Mix (Perfect Real Time; Takara) was used for reverse transcription of 1 μg of RNA into cDNA. Quantitative PCR was performed with SYBR-Green premix Extaq (Takara) and detected by a Real-Time PCR System (CFX96; Bio-Rad). Primers: GAPDH (forward: 5′-TTGTGATGGGTGTGAAC CACGAGA-3′ and reverse: 5′-GAGCCCTTCCACAATGCCAAAGTT -3′), GPR37 (*Mus musculus*; 5′-ACCGGACACAATCTATGTTTTGG-3′ and reverse: 5′-TCTTCCGAGCAGTCACTAGAG-3′) and opto-GPR37 (forward: 5′-TGACAACGAGTACACCACGG-3′ and reverse: 5′-GCTTCGTCGCA ATGAGTTCC-3′). Following PCR amplification, the derivative melting curve analysis was conducted to confirm the specificity of the PCR. Ct values were converted to relative quantification using the 2^−ΔΔCt^ method.

### Behavioral Tests

The open-field test was conducted in an open-field chamber (44 × 44 cm^2^). Mice were placed in the cages and allowed to move for a few minutes freely. Locomotor activity was recorded with a video camera set on the ceiling above the arena and connected to an automated video tracking system (EthoVision system, Noldus). On the first day, mice were stimulated with the light (473 nm, 20 ms, 20 Hz, 10 mW) for 5 min according to the protocol of our previous study (Li et al., 2015, 2016). Total distance and residence time in the central area of the open field were recorded. On the third day, after the light stimulation for 5 min (473 nm, 20 ms, 20 Hz, 10 mW) the mice were again put into the open field, and the total distance and residence time in the central area of the open field were recorded. For spontaneous alternation test in the Y-maze, the mice were placed at the end of one arm and subjected to blue light for 5 min (473 nm, 20 ms, 20 Hz, 10 mW). The sequence of animal entries to each arm and the number of entries during 5 min were traced. Alternation was defined as the continuous entry into three arms (such as 1, 2, 3 or 1, 3, 2), and the maximum alternation—as two entries fewer than the total number of arm entries. We then calculated the percentage of entries (i.e., the actual alternation/maximum alternation × 100%). The number of entries was recorded to estimate the locomotor activity.

### Statistics

All experiments were performed by a single investigator who was blind to the treatments of the experimental animals. Statistical analyses were carried out with GraphPad software (version 6.0). The data are presented as mean ± SEM. Student’s *t*-test was used to compare the effect of opto-GPR at a single time point (Figures 2B–G, 4D). One-Way ANOVA with Dunnett’s *post hoc* test was used to analyze the effect of opto-GPR on signaling at multiple time points (Figure 2H). Two-Way ANOVA with Tukey’s *post hoc* test was used to analyze the effect of opto-GPR37, time course and their interaction (Figures 3D, 4B, 5A–F). *P* < 0.05 (*), *p* < 0.01 (**) and *p* < 0.001 (***) were considered as significant.

**Figure 1:**
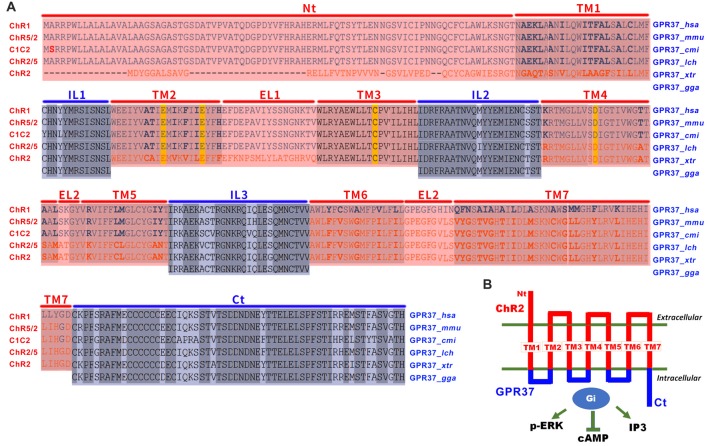
The design of opto/channelrhodopsin 2 (ChR2)-GPR37 chimera. **(A)** To create the ChR2-GPR37 chimera, we used extracellular and transmembrane domains (TM) of ChR2 (synthetic construct, ABO64386.1) as previously defined by sequence alignment of channelrhodopsin family (Inaguma et al., 2015). We then used the intracellular loop (IL) and C terminus (Ct) of GPR37 (Homo sapiens, NP_005293.1) as defined by the alignment of GPR37 sequence from different species that share conserved structure of seven-helix TM in the NCBI database (cd15127: 7tmA_GPR37). We constructed a fusion gene encoding a chimera (ChR2-GPR37) by fusing the extracellular loops (EL) 1, 2 and 3 and the N terminus (Nt) of ChR2 with the ILs and C terminus of GPR37. Blue: ILs and C terminus of GPR37; red: N terminus, EL and TM of ChR2. *Homo sapiens* (*hsa*), *Mus musculus* (*mmu*), *Callorhinchus milii* (*cmi*), *Latimeria chalumnae* (*lch*), *Xenopus tropicalis* (*xtr*) and *Gallus gallus* (*gga*). **(B)** Schematic illustration of the opto/ChR2-GPR37 chimera comprising the N terminus, extracellular and TM of ChR2 fused with the intracellular and C terminal parts of GPR37.

**Figure 2:**
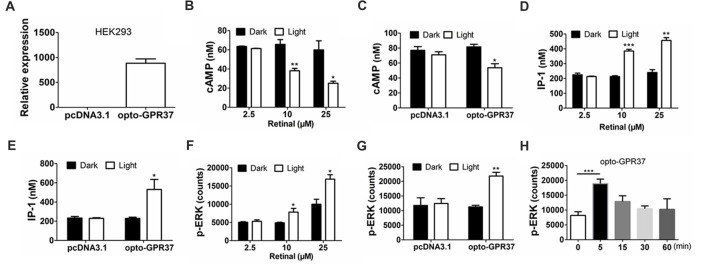
Expression and light activation of opto-GPR37 signaling in HEK293 cells. **(A)** Expression of opto-GPR37 in HEK293 cells was determined by qPCR using the primers targeting the intracellular part of human GPR37 used in the opto-GPR37 chimera. **(B)** Light activation of opto-GPR37 decreased the cAMP accumulation with different concentrations of the retinal in three independent experiments (*p* = 0.0077, *p* = 0.0233, Student’s *t*-test). **(C)** Light activation of opto-GPR37 decreased the cAMP accumulation with 25 μM retinal (*N* = 3/group, *p* = 0.0124, Student’s *t*-test). **(D)** Light activation of opto-GPR37 led to the accumulation of IP-1 with different concentrations of the retinal (*p* = 0.0002, *p* = 0.0013, Student’s *t*-test). **(E)** Light activation of opto-GPR37 led to the accumulation of IP-1 with 25 μM retinal (*N* = 3/group, *p* = 0.0424, Student’s *t*-test). **(F)** Light activation of opto-GPR37 enhanced the phosphorylation of ERK with different concentrations of the retinal (*p* = 0.0489, *p* = 0.0191, Student’s *t*-test). **(G)** Light activation of opto-GPR37 enhanced the phosphorylation of ERK with 25 μM retinal (*p* = 0.0015, Student’s *t*-test). **(H)** Kinetic increases in p-ERK in response to the light (0, 5, 15, 30, 60 min; *p* = 0.0007; One-Way ANOVA with Dunnett’s *post hoc* test). *p < 0.05, **p < 0.01, ***p < 0.001.

**Figure 3:**
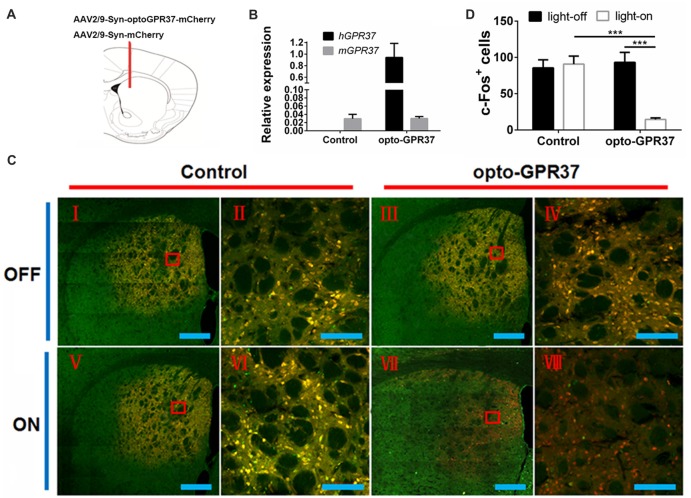
Expression and light action of opto-GPR37 function in the striatum. **(A)** Top panel: schematic diagram (taken from the 4th edition of Paxinos and Franklin’s the Mouse Brain in Stereotaxic Coordinates) of the injection site. **(B)** Expression of opto-GPR37 in the striatum was determined by qPCR using the primers targeting the intracellular part of human GPR37 used in the opto-GPR37 chimera. The expression level of opto-GPR37 in the striatum was significantly higher than the endogenous mouse GPR37 (after normalization with the internal control GAPDH). **(C)** Light activation of opto-GPR37 for 15 min reduced the c-Fos expression (almost all c-Fos^+^ cells merged with mCherry^+^ cells, as indicated by yellow) in the striatum of freely moving animals; c-Fos (green), mCherry (red), merge (yellow), scale bar = 400 μm (I, III, V, VII) or 100 μm (II, IV, VI, VIII). **(D)** Quantitative analysis showed that light stimulation of opto-GPR37 has markedly reduced the level of c-Fos- and mCherry-double positive cells (merged c-Fos^+^/mCherry^+^ cells in yellow) in the striatum, as compared with the control (mCherry) group (*N* = 5/group, *p* = 0.0005, *p* = 0.0004; Two-Way ANOVA with Tukey’s *post hoc* test, *N* = number of animals/group). ***p < 0.001.

**Figure 4:**
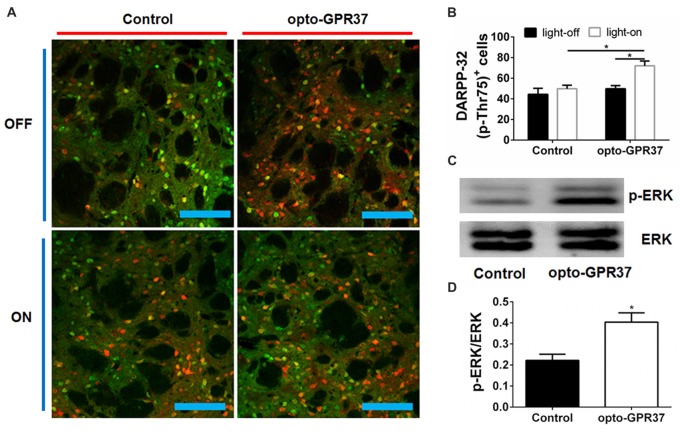
Light activation of opto-GPR37 increased DARPP-32 (p-Thr75) in the striatum. **(A)** Analysis of DARPP-32 (p-Thr75) immunoreactivity by fluorescence immunohistochemistry after the light activation of opto-GPR37 for 10 min. DARPP-32 (p-Thr75) phosphorylation (green), mCherry (red), scale bar = 100 μm. **(B)** Quantitative analysis of DARPP-32 (p-Thr75)-positive and mCherry-positive cells (i.e., DARPP-32-pThr75^+^/mCherry^+^ merged cells in yellow) after light stimulation of opto-GPR37 in the striatum of the mCherry- and opto-GPR37-transfected mice (*N* = 5/group, *p* = 0.0123, *p* = 0.0121; Two-Way ANOVA with Tukey’s *post hoc* test). **(C)** Western blot analysis shows that in response to light stimulation p-ERK level increased in the striatum of opto-GPR37-transfected mice, as compared with the control (mCherry-transfected striatum). **(D)** Quantitative analysis of p-ERK level after light stimulation in the striatum of the control (mCherry-transfected) and opto-GPR37-transfected striatum by Western blot with ImageJ software (*N* = 4/group, *p* = 0.0140, Student’s *t*-test, *N* = number of animals/group). *p < 0.05.

**Figure 5:**
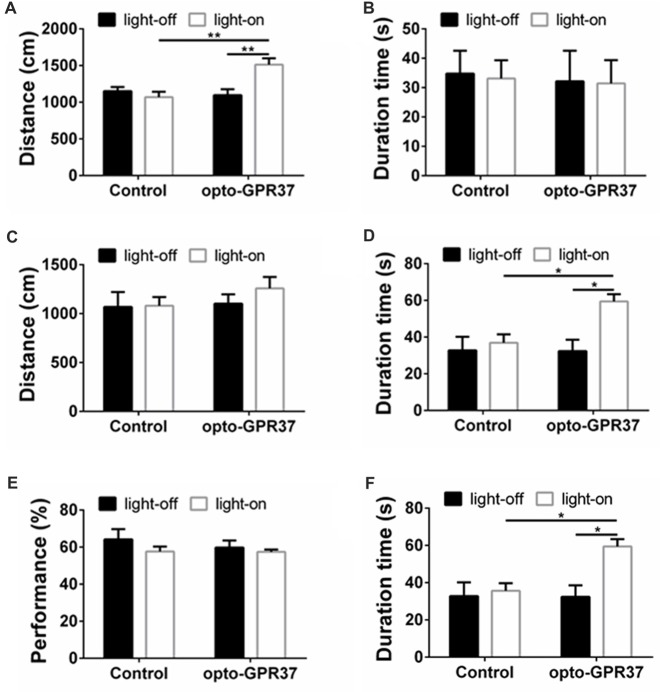
Light illumination of opto-GPR37 improved the locomotor activity and anti-anxiety behavior in mice.** (A)** Activation of opto-GPR37 in dorsal medial striatum (DMS) increased the total distance in comparison with the control (*N* = 7/group, *p* = 0.0019, *p* = 0.0036; Two-Way ANOVA with Tukey’s *post hoc* test). **(B)** Activation of opto-GPR37 for 5 min did not affect the residence time in the central area of the open field (*N* = 7/group, Two-Way ANOVA with Tukey’s *post hoc* test). **(C)** After the light stimulation, distance covered by opto-GPR37 mice was not different between the opto-GPR37 and control groups (*N* = 7/group, Two-Way ANOVA with Tukey’s *post hoc* test). **(D)** The opto-GPR37 mice spent more time in the central area of the open field than the control mice after the light stimulation (i.e., delay effect of opto-GPR37; *N* = 7/group, *p* = 0.0287, *p* = 0.0111; Two-Way ANOVA with Tukey’s *post hoc* test). **(E)** Activation of opto-GPR37 did not affect working memory in the spontaneous alternation test using Y-maze (*N* = 7/group, Two-Way ANOVA with Tukey’s *post hoc* test). **(F)** Induction of opto-GPR37 improved the number of entries in the Y-maze (*N* = 7/group, *p* = 0.0191, *p* = 0.0191; Two-Way ANOVA with Tukey’s *post hoc* test, *N* = number of animals). *p < 0.05, **p < 0.01.

## Results

### Light Activation of opto-GPR37 Induces cAMP, p-ERK and IP-3 Signaling in HEK293 Cells

By replacing the extracellular and transmembrane portions of GPR37 with the corresponding parts of ChR2, we designed a chimeric protein opto-GPR37 to trigger GPR37 signal transduction (Figure 1, Supplementary Figures S1, S2). We transfected the plasmid with opto-GPR37 sequence into human embryonic kidney cells (HEK293-opto-GPR37; Figure 2A). We added different (1–25 μM) concentrations of all-*trans*-retinal to medium 48 h after the transfection. In response to blue light stimulation of opto-GPR37 the cAMP level was dramatically decreased in the presence of 10 μM and 25 μM retinal in HEK293 cells as compared with the dark condition (Figure 2B). We further analyzed cAMP level in HEK293 cells transfected with opto-GPR37 and in the cells transfected with the control plasmid. We found that activation of opto-GPR37 indeed decreased the concentration of cAMP in the presence of 25 μM retinal, whereas there were no changes in control samples (Figure 2C). Also, there was no difference between the opto-GPR37 and the control under the dark condition (Figure 2C). The reduction of cAMP by light activation of opto-GPR37 is consistent with previous transgenic studies indicating that GPR37 couples to G_i_ protein signaling pathway.

Prosaptide can bind to GPR37 and increase ERK phosphorylation (p-ERK) in HEK293 cells. We also observed that 60-s light stimulation of opto-GPR37 in HEK293 cells increased p-ERK level in the presence of 10 μM or 25 μM retinal. In comparison with the control, light stimulation of HEK293-opto-GPR37 increased phosphorylation level of ERK by about 2-fold (Figures 2F,G). However, no significant difference was revealed under the dark condition (Figures 2F,G). Thus, opto-GPR37 recruits two parallel GPR37 signaling pathways, namely G_i_-cAMP and ERK signaling in HEK293 cells. To further characterize the kinetic changes of p-ERK in response to light, we stimulated opto-GPR37 for 60 s and analyzed the p-ERK levels at 0, 5, 15, 30 and 60 min after the stimulation. We observed a rapid increase in p-ERK (5 min) in response to light stimulation of opto-GPR37 (Figure 2H), but the p-ERK quickly returned to the baseline level given that p-ERK levels at these time points were not different from the baseline (Figure 2H).

Also, we measured the IP-1 production, a degradation product of IP-3 (inositol-1,4,5-triphosphate). Interestingly, we found that IP-1 level was strikingly induced by the light stimulation, as compared with the dark condition (Figures 2D,E).

### Light Activation of opto-GPR37 Reduces c-Fos Expression and Enhances DARPP-32 (Thr75) and ERK Phosphorylation in the Striatum of Freely Moving Animals

To assess whether the light activation of opto-GPR37 triggers similar intracellular signaling pathways in intact animals, we injected AAV2/9-syn-opto-GPR37-mCherry or AAV2/9-syn-mCherry (control) viruses into the DMS (Figures 3A,B). We assessed functional consequence of the reduced cAMP signaling elicited by the light activation of opto-GPR37 in the striatum by evaluating DARPP-32 (Thr75) phosphorylation and c-Fos expression. DARPP-32 (DA and cyclic AMP-regulated phosphoprotein, 32 kDa) is a cytosolic protein highly present in medium-sized spiny neurons of the striatum and is subjected to modulation by cAMP level. Decreased cAMP production and reduced PKA activity following A_2A_R blockade (Lindskog et al., 2002; Hsu et al., 2009), as well as A_2A_R KO (Shen et al., 2013) or activation of the D2 receptor (Svenningsson et al., 2004) not only reduced DARPP-32 phosphorylation at Thr34 but also increased DARPP-32 phosphorylation at Thr75. Consistent with this prediction, we found that in response to the light stimulation for 10 min, the brain slices of opto-GPR37 showed stronger fluorescence intensity of DARPP-32 (pThr75), as compared with the control (Figures 4A,B).

Similarly, the reduced cAMP level can also reduce Jun-B phosphorylation resulting in reduced c-Fos expression. To determine the effect of opto-GPR37 activation on striatal c-Fos expression we stimulated opto-GPR37 or the control for 15 min. Ninety minutes after the light stimulation the mouse striatum was dissected and processed for immunofluorescence detection of c-Fos expression. Significant basal expression of c-Fos^+^ signals was detected in the striatum of the control mice, particularly in the dorsomedial part (Figures 3C,D). Consistent with our prediction, optical stimulation of opto-GPR37 reduced c-Fos^+^ expression in the striatum, as compared with the control (Figures 3C,D). These results on DARPP-32 (pThr75) and c-Fos expression are consistent with the finding that stimulation of opto-GPR37 caused a decrease of cAMP indicating the GPR37-G_i_ protein coupling signaling.

Moreover, we also examined whether opto-GPR37 produces a similar effect on ERK signaling since the previous study also revealed that KO or activation of GPR37 respectively reduced or improved the phosphorylation of ERK. To characterize p-ERK pattern in the striatum, we had to collect the focal areas of DMS surrounding light-stimulated zone (<10% striatum) to enhance the ability to detect p-ERK by Western blot analysis. To do so, we injected blue dye through the same cannula after the light stimulation and used blue dye as the indicator of the virus injection and light stimulation site to dissect out and collect the striatum with blue staining. With this focal dissection technique and with the pooled striatum (3–5 mice per sample) we showed that the light activation of opto-GPR37 for 10 min (4 samples from 15 to 20 mice) increased the phosphorylation level of ERK compared with the control as measured by Western Blotting (Figures 4C,D, Supplementary Figure S4, *p* = 0.0140). This increase in p-ERK was transient, and p-ERK returned to the baseline level because other time points (5 min, 30 min, one pooled sample for each time point) did not differ from the control (data not shown). Thus, the light activation of opto-GPR37 triggered a rapid but transient ERK signaling.

### Light Stimulation of opto-GPR37 Increases Locomotor Activity and Induces Anti-Anxiety-Like Behavior

To determine behavioral response to the light activation of opto-GPR37, we used open field and Y-maze tests to assess their motor activity and anxiety-like behavior in response to opto-GPR37. The AAV2/9-syn-opto-GPR37-mCherry and the control virus were injected into the DMS of C57B/L6 mice. Three weeks after the injection we measured spontaneous locomotor activity using open field test. In response to blue light stimulation, the mice transfected with opto-GPR37 showed a marked increase in total distance, as compared with the control (Figure 5A). This increased locomotor activity was observed only during the light stimulation phase (Figure 5A). We found no difference in the total distance at the post-stimulation period (Figure 5C). Besides, the residence time in the central area was not different among four groups during the stimulation phase (Figure 5B). Moreover, after activation of opto-GPR37 for 5 min (in the absence of light stimulation) the opto-GPR37 mice stayed for a more extended time in the central area of the open-field arena compared with controls (Figure 5D). Thus, opto-GPR37 activation may produce the delay effect on anxiety-like behavior.

We further used Y-maze-based spontaneous alternation test to assess working memory in response to the light activation of opto-GPR37. The results showed no difference in working memory performance between the mice transfected with opto-GPR37 and the control (Figure 5E). However, we noted that a total number of entries into the maze arms was increased in opto-GPR37 mice compared with the control, consistent with the above open-field results (Figure 5F). Thus, activation of opto-GPR37 did not affect working memory despite its enhancement of motor activity.

To exclude the potential cellular damage caused by the light stimulation or overexpression of opto-GPR37, we examined the striatum for possible tissue damage using double strain DNA break by TUNEL staining (Supplementary Figure S3). We stimulated the opto-mCherry and opto-GPR37 groups for 30 min. Although visible TUNEL signals were *observed* in the DNase I-treated positive control (Supplementary Figures S3A,E), almost no positive signals were revealed in the striatal sections (opto-mCherry and opto-GPR37) following the light stimuli with the same settings and intensities (Supplementary Figures S3B,D,F,H). Also, few signals were detected in the opto-GPR37 group (with no light stimuli; Supplementary Figures S3C,G).

## Discussion

This study is, to the best of our knowledge, the first demonstration of the opto-orphan GPCR application to bypass the endogenous ligand and trigger orphan GPCR signaling for probing its function. Using GPR37 as an example, we designed and characterized the opto-GPR37 to demonstrate the specificity of GPR37 signaling (i.e., cAMP and ERK signaling) and uncover novel aspects of GPR37 signaling (i.e., IP-3 signaling). Light activation of opto-GPR37 permits the causal analysis of GPR37 activity in the defined cells and behavioral responses of freely moving animals. Moreover, given the evolutionarily conserved seven-helix transmembrane structure of orphan GPCRs and also well-defined channelrhodopsin family (such as ChR2), we propose that the opto-GPR37 technique represents an entirely new approach for deorphanization of GPCRs in freely moving animals by optogenetics.

### Light Activation of opto-GPR37 Triggers Specific GPR37 Signaling in Defined Cells of Freely Moving Animals With New Insights

The opto-GPR37 (a chimeric ChR2-GPR37 protein) retains the extracellular and TM of ChR2 to confer light responsiveness to bypass the required endogenous ligand and trigger GPR37 signaling in defined cells of freely moving animals. Critically, the extracellular and TM of ChR2 are fused with the IL of GPR37 to elicit specific GPR37 signaling. Thus, the critical aspect of opto-GPR37 development is the confirmation of specificity of the signaling cascade consistent with the previous studies by transgenic overexpression and genetic KO of GPR37. The following experimental findings support this conclusion: (I) Consistently with the research that overexpression of GPR37 resulted in the decrease of cAMP, we observed a rapid decline of cAMP level following light stimulation for just 60 s in cultured HEK293 cells. (II) Similarly, reduced c-Fos expression in the striatum following light activation of opto-GPR37 is consistent with the G_i_ coupling of GPR37, because reduced cAMP-PKA can lead to a decrease in c-Jun/Jun-B phosphorylation (Abate et al., 1991). (III) Increased DARPP-32 phosphorylation at Thr75 has been demonstrated by reduced cAMP production and PKA activity following A_2A_R blockade (Lindskog et al., 2002; Hsu et al., 2009), A_2A_R KO (Shen et al., 2013) or D2 receptor activation (Svenningsson et al., 2004). The increased phosphorylation of Thr75 of DARPP-32 by light activation of opto-GPR37 agrees with the reduced cAMP-PKA signaling at Thr34 and increased phosphorylation of DARPP-32 (Thr75; Svenningsson et al., 2004). (IV) Previous studies showed that overexpression of GPR37 leads to an increase in p-ERK, whereas an absence of GPR37 impairs the phosphorylation of Akt and ERK2 (Marazziti et al., 2011). Consistent with these findings, we detected the increased ERK phosphorylation in response to light activation of opto-GPR37 in cultured HEK293 cells and the striatum. (V) Consistent with the GPR37-KO study showing that deletion of GPR37 reduces locomotor activity (Marazziti et al., 2004; Lopes et al., 2015) and enhances DA uptake with the reduced DA content in the striatum (Marazziti et al., 2004), we found that activation of opto-GPR37 in DMS enhances motor activity, possibly through modulation of the striatal DA content. Collectively, these findings strongly support that light activation of opto-GPR37 triggers specific signaling pathway of GPR37, as shown by genetic studies at the signaling (cAMP and p-ERK), neurochemical (c-Fos and DRAPP-32) and behavioral (locomotor activity) levels.

Our analysis uncovered that light activation of opto-GPR37 increased the level of IP-1 in HEK293 cells showing that GPR37 may also couple with the previously unknown G_q_ signaling. Moreover, opto-GPR37-induced increase in the residence time in the central area of the open field chamber indicates the possible involvement of GPR37 in anxiety-like behavior. Last, given that GPR37 is emerging as a potential therapeutic target for PD for its ability to prevent dopaminergic neurotoxicity, opto-GPR37 can be used to explore this potential in the defined cell types in PD.

### Opto-XR Represents a Novel Approach for Deorphanization of GPCRs

Despite the intensive efforts and significant progress in deorphanization of GPCRs, ≈100 GPCRs are still classified as orphan receptors without identified endogenous ligands and unknown physiological functions (Wise et al., 2004; Allen and Roth, 2011; Civelli, 2012; Davenport et al., 2013). Exploration of orphan GPCR functions has been limited to the genetic studies by overexpression and gene silencing (Levoye and Jockers, 2008). These genetic approaches lack temporal resolution and have hampered the efforts to decipher orphan GPCR functions under various pathophysiological conditions, such as cancer, and disorders of neurodegenerative and autism spectra (Fujita-Jimbo et al., 2012; Tang et al., 2012; Ahmad et al., 2015). The current reverse pharmacology strategy with high-content screening in cultured cells with the second messengers as readouts may not provide suitable conditions for the discovery of the remaining orphan GPCRs. Many GPCRs and some endogenous ligands are restricted to defined cell types and thus activate their signaling cascades in a local (brain-region specific) manner (Chung et al., 2008; Katritch et al., 2013; Kenakin, 2013). Since many of the orphan GPCR signaling pathways might engage in higher brain functions (Wise et al., 2004; Chung et al., 2008; Allen and Roth, 2011), deorphanization of the remaining orphan GPCRs would require strategies to: (a) bypass the unknown ligand as the source for orphan GPCR signaling; (b) activate orphan GPCR signaling in local environments; and (c) use functional readouts beyond the secondary messenger levels (e.g., elements of addictive behaviors).

Optogenetic (“opto-orphanGPCR”) approaches (Kim et al., 2005) may fill in this gap with some distinct advantages, namely the possibility of cell- and brain region-specific orphan GPCR activation and functional detection beyond second messenger levels (e.g., therapy-related behaviors). Thus, using the AAV-mediated targeted expression of opto-orphanGPCR chimeras in distinct brain structures, the opto-orphanGPCR approach can reveal orphan GPCR signaling on a physiologically relevant timescale (Boyden et al., 2005; Kim et al., 2005; Airan et al., 2009). It could also permit spatiotemporally precise control of orphan GPCR signaling in freely behaving animals. Opto/ChR2-orphanGPCR approaches allow direct identification of specific orphan GPCRs that modulate behaviors of freely moving animals. As many neuropsychiatric disorders are primarily behavioral disorders, this ability of the opto/ChR2-GPR37 approach to producing cell-type/brain region-specific orphan GPCR activation and to use therapeutically-related behaviors as a functional readout will greatly facilitate the identification of the remaining orphan GPCRs affecting neuropsychiatric behaviors. Because multiple alignment of homologous sequences of different species well defines the seven-helix transmembrane structure (“boundary”) of both channelrhodopsin family and orphan GPCRs, the simple design of opto-orphanGPCR (in particular, ChR2-orphanGPCR) chimera used in this study can be readily applied to many other orphanGPCRs to facilitate elucidation of their signaling and behavioral responses in intact animals.

## Author Contributions

J-FC and WZ conceived and designed the experiments and wrote the article; WZ, YL, JY, ZW, MW, XC and FL performed the experiments; WZ, JZ, SV and ZL analyzed the data; WZ, WG, JZ, YG and BW contributed reagents/materials/analysis tools; SV also contributed to the manuscript text edition.

## Conflict of Interest Statement

The authors declare that the research was conducted in the absence of any commercial or financial relationships that could be construed as a potential conflict of interest.
