# Supplementary material for: Spatiotemporal Control of GPR37 Signaling and Its Behavioral Effects by Optogenetics
Source: Front Mol Neurosci. 2018 Mar 28;11:95. doi: 10.3389/fnmol.2018.00095 (PMC5882850; doi:10.3389/fnmol.2018.00095)
Supplement: Supplementary file 1 [file Image_1.PDF]

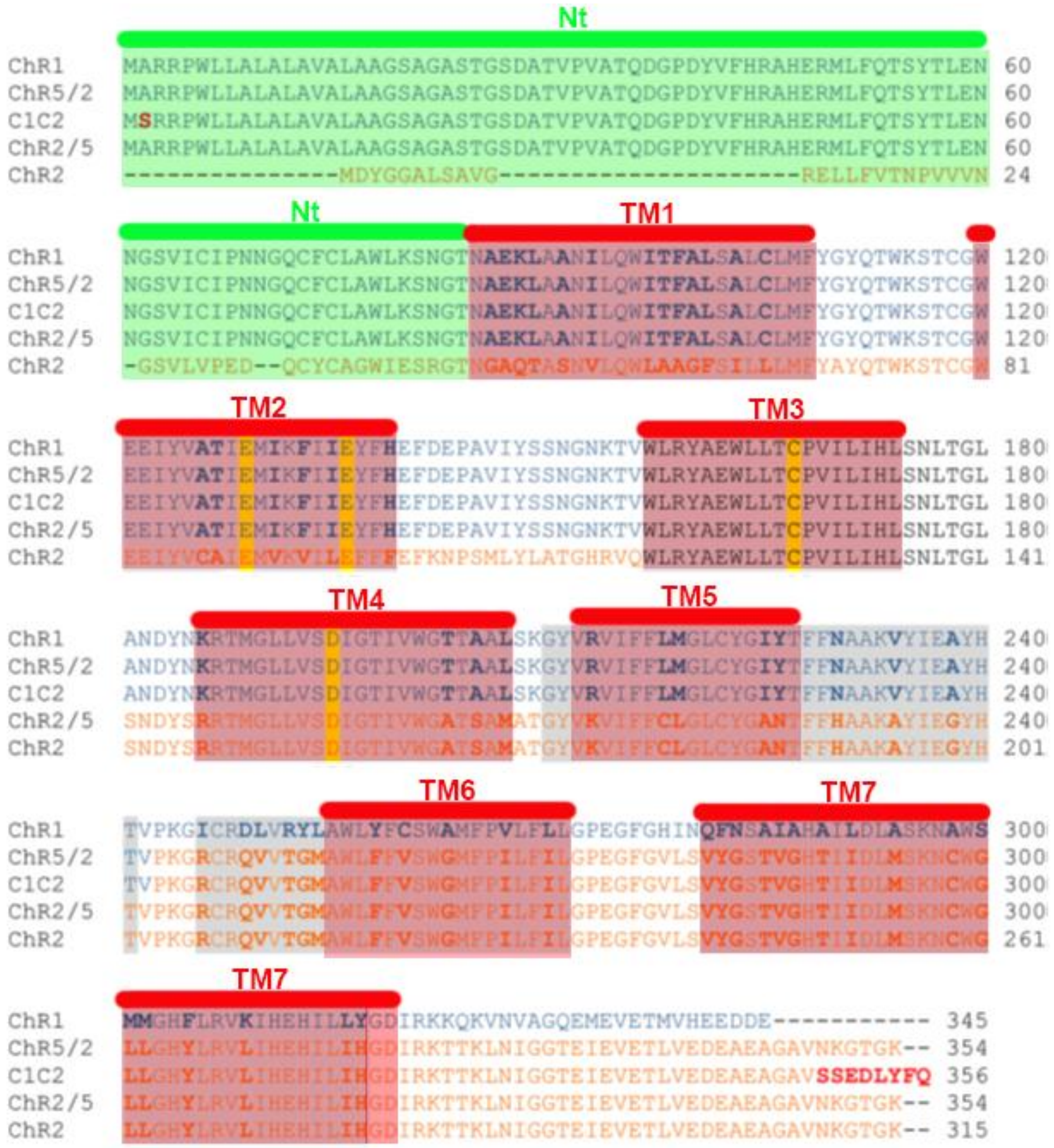

Figure S1. Sequence alignment of ChR1, ChR2 and the chimeras (ChR5/2, ChR2/5, and C1C2). The figure was quoted from the research (Inaguma et al., 2015). Nt: N terminus (green); TM: transmembrane domains (red).
