# Supplementary material for: Spatiotemporal Control of GPR37 Signaling and Its Behavioral Effects by Optogenetics
Source: Front Mol Neurosci. 2018 Mar 28;11:95. doi: 10.3389/fnmol.2018.00095 (PMC5882850; doi:10.3389/fnmol.2018.00095)
Supplement: Supplementary file 2 [file Image_2.PDF]

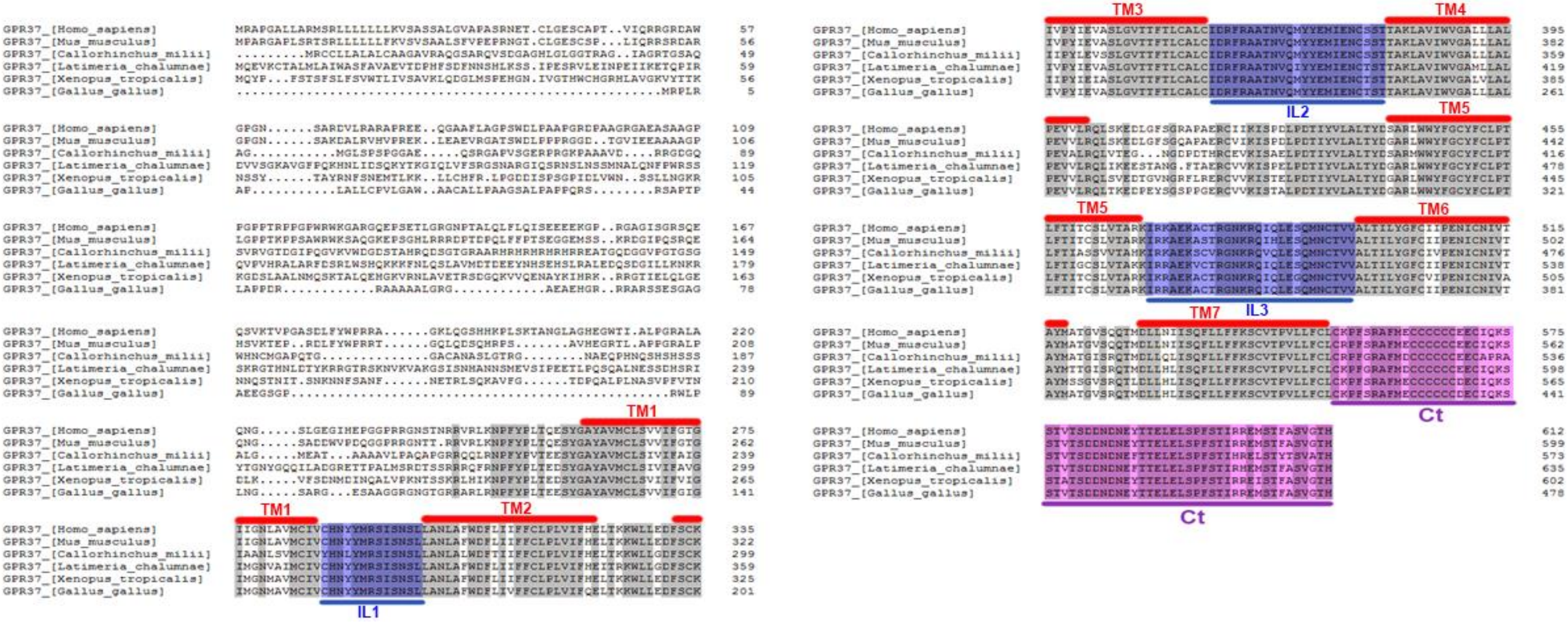

Figure S2. Sequence alignment of GPR37 from different species (*Homo sapiens*, *Mus musculus*, *Callorhinchus milii*, *Xenopus tropicalis*, and *Gallus gallus*). TM: transmembrane domains (Red); IL: intracellular loop (Dark blue); Ct: C terminus (purple).
