# Supplementary material for: Spatiotemporal Control of GPR37 Signaling and Its Behavioral Effects by Optogenetics
Source: Front Mol Neurosci. 2018 Mar 28;11:95. doi: 10.3389/fnmol.2018.00095 (PMC5882850; doi:10.3389/fnmol.2018.00095)
Supplement: Supplementary file 3 [file Image_3.PDF]

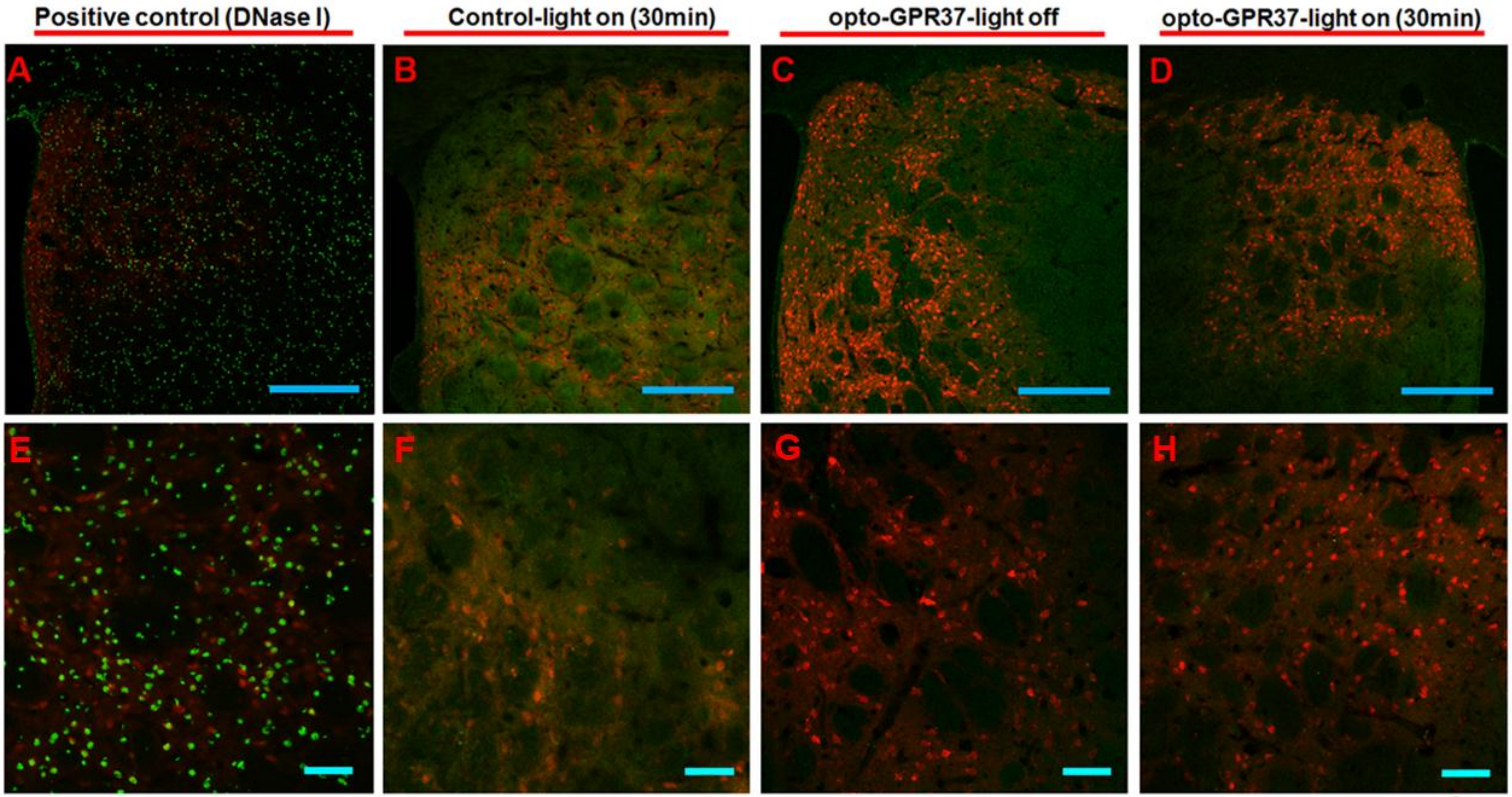

Figure S3. Analysis of TUNEL staining in the striatum after the light activation of opto-GPR37 (473 nm,10 mW, 20 ms, 20 HZ) for 30 min. After the light stimulation of opto-GPR37 for 30 min, no positive TUNEL signals in the striatal slices of three groups were detected (B-D; F-H). Visible TUNEL signals were detected in the DNAse I-treated positive control (A and E); mCherry (red), TUNEL (green); upper panel: scale bar = 200 μm, lower panel: scale bar = 50 μm.
