# Supplementary material for: Spatiotemporal Control of GPR37 Signaling and Its Behavioral Effects by Optogenetics
Source: Front Mol Neurosci. 2018 Mar 28;11:95. doi: 10.3389/fnmol.2018.00095 (PMC5882850; doi:10.3389/fnmol.2018.00095)
Supplement: Supplementary file 4 [file Image_4.PDF]

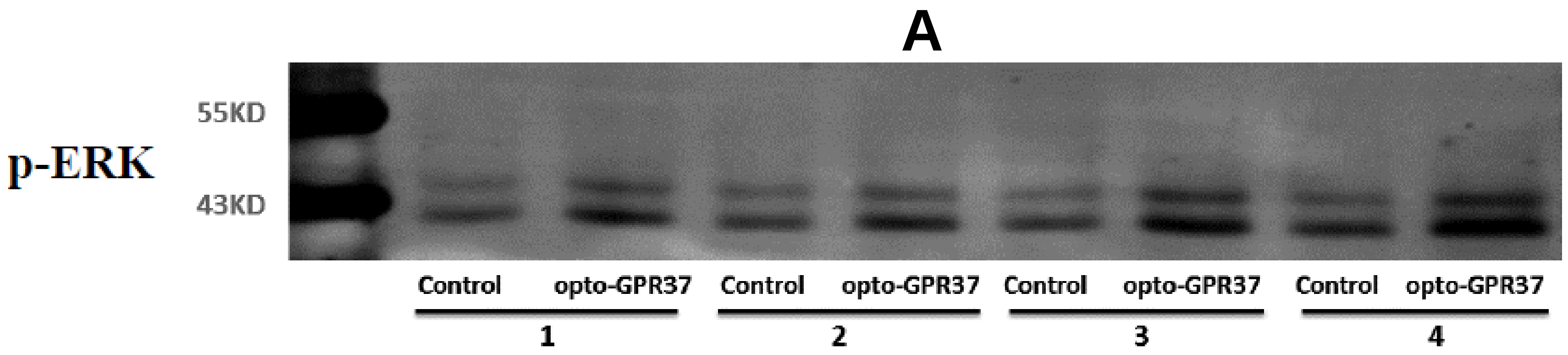


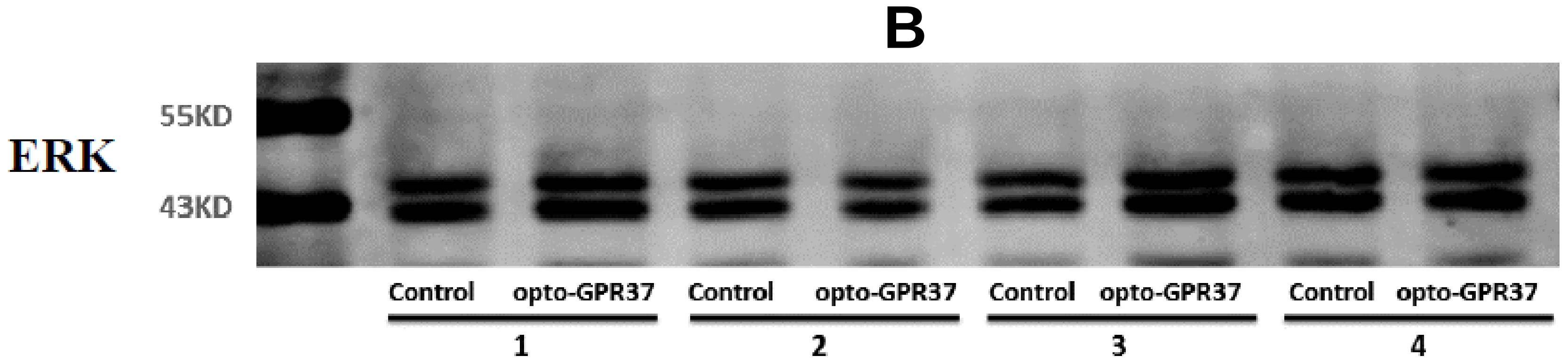


**Figure S4:** The original images of Western Blot analysis of p-ERK (A) and total ERK (B) after light activation of ChR2-GPR37. The p-ERK and total ERK levels for the control (mCherry-transfected) and opto-GPR37-transfected striatum were analyzed by WB for four samples (labeled as “1”, “2”, “3”, and “4”), with each sample being pooled from 3-5 mice (see the Methods for the details). Ten minutes after light stimulation, the level of p-ERK in the opto-GPR37 group increased in comparison with the control group (N = 4 / group, N = number of samples). The lines from the sample “4” (A, B) were used in the Figure 4C.
